# The Effect of Treatment With Clear Aligners Versus Fixed Appliances on Oral Health-Related Quality of Life in Patients With Severe Crowding: A One-Year Follow-Up Randomized Controlled Clinical Trial

**DOI:** 10.7759/cureus.25472

**Published:** 2022-05-30

**Authors:** Samer T. Jaber, Mohammad Y Hajeer, Ahmad S Burhan, Youssef Latifeh

**Affiliations:** 1 Department of Orthodontics, University of Damascus Faculty of Dentistry, Damascus, SYR; 2 Department of Internal Medicine, University of Damascus Faculty of Medicine, Damascus, SYR

**Keywords:** psychological disability, physical disability, psychological discomfort, physical pain, functional limitation, oral health impact profile, crowding, oral health-related quality of life, clear aligners, class i malocclusion

## Abstract

Objective

To compare the level of oral health-related quality of life (OHRQoL) between patients receiving clear aligners or fixed appliances within one year of follow-up using Oral Health Impact Profile 14 (OHIP-14), a validated self-administered questionnaire.

Materials and methods

A single-centered, two-arm parallel-group randomized controlled clinical trial was conducted on 36 adult patients (19 females, 17 males; age range: 18 to 25 years) who had severe crowding and require orthodontic treatment with first premolars extraction. The patients were equally and randomly divided into two groups: The clear aligners (CA) group and the fixed appliances (FA) group. OHRQoL was assessed using the OHIP-14 tool at various times during comprehensive orthodontic therapy: baseline (T0), one week (T1), two weeks (T2), one month (T3), 6 months (T4), and 12 months (T5) after starting the active orthodontic treatment. Mann-Whitney U test or Friedman test were used to detect significant differences. The level of significance was set at 5%.

Results

All of the selected patients entered the statistical analysis stage. There were no significant differences between the CA and FA groups for the psychological discomfort, psychological disability, social disability, and handicap (P˃0.05) at almost all assessment times. For the functional limitation, physical pain, physical disability, and the overall score, there were significant differences between the studied groups (P˂0.05), with the FA group having higher mean scores than the CA group in all of the assessment times.

Conclusion

Patients' treatment with clear aligners has less impact on OHRQoL than those treated using conventional fixed appliances during the first year of treatment.

## Introduction

Malocclusion is a deviation from an ideal occlusion or an accepted societal norm. Many abnormalities are within the normal biologic variation among individuals [1]. Dental malocclusion is very common among children and adolescents worldwide [2]. It's associated not only with oral disorders but has a strong impact on a patient's psychological, social, and functional health [3,4] and rating of intelligence [5]. These psychological, social, and functional aspects are referred to as oral health-related quality of life (OHRQoL). Modern orthodontic treatments are striving to offer patients a comfortable and pleasant treatment journey [6]. Therefore, several studies have emerged recently to focus on patients' centered outcomes during a wide variety of orthodontic interventions [7-11].

A wide range of orthodontic techniques are available, and the choice depends on the type of malocclusion, the existence of associated disorders, and the patients' esthetic and functional demands [12]. Fixed appliances are considered the most common technique used in the treatment of adolescents and adults [13]. Several studies have investigated the effect of orthodontic treatment with fixed appliances on the patients' quality of life [14,15], whereas other studies have focused on their short- and long-term impact on the OHRQoL [16,17]. Palomares et al. reported that orthodontic treatment significantly improved the OHRQoL when comparing treated patients with non-treated subjects [15]. Jawaid and Qadeer [18] and Mansor et al. [19] found a significant deterioration in the OHRQoL 24 hours after the onset of fixed appliances treatment. Costa et al. concluded that fixed orthodontic appliances have significantly worsened the OHRQoL in treated patients compared with children without any malocclusion or orthodontic appliances after three months of the active treatment [17].

Clear aligners are the most fast-growing technique in the orthodontic field [20]. Effective performance of clear aligners comparable with or even superior to the fixed appliances was demonstrated in the latest studies considering the clinical complexity of the cases [21]. Patients who choose to be treated with clear aligners often seek appliances that have fewer impacts on their daily lives. They are willing to incur greater costs to acquire an orthodontic treatment with less negative effects on their quality of life [22]. They also choose this technique due to its superior esthetics [23]. Only a few studies in the literature compare patient experiences and treatment effects between clear aligners and fixed appliances.

Shalish et al. compared the adaptation to fixed appliances and clear aligners provided by Invisalign® in adult patients using a self-designed OHRQoL questionnaire. During the first week of treatment, clear aligners patients reported severe pain more than the fixed appliance group. Similar levels of general activity disturbances and oral dysfunction were reported for both of the studied groups, whereas the clear aligners group reported lower oral symptoms, and the fixed appliances group reported significantly higher concerns about eating difficulties [24]. Flores-Mir et al. used Oral Impacts on Daily Performance (OIDP) and Patient Satisfaction Questionnaire (PSQ) to assess patients' satisfaction and quality of life among adults treated with fixed appliances or clear aligners provided by Invisalign® immediately after completion of their treatment. Similar satisfaction outcomes were found for almost all the dimensions investigated in both groups. However, concerning eating and chewing, almost half of the clear aligners group reported 100% satisfaction, compared to only 24% in the fixed appliances group [25]. A recent cohort study [26] assessed the OHRQoL between patients receiving clear aligners and fixed appliances in the first two weeks of treatment using oral health impact profile-14 (OHIP-14). This study showed that both groups had their greatest OHIP-14 scores (i.e., the worst quality of life) on the first day and gradually decreased after that, and the OHRQoL was generally higher in the clear aligners group. A recent systematic review [6] indicated that the effect of clear aligners treatment on OHRQoL compared to the treatment with fixed appliance treatment is still inconclusive, and future studies using appropriate measuring tools are needed.

Reviewing the available literature indicates no randomized controlled trial (RCT) investigating the effects of clear aligners therapy on OHRQoL compared to fixed appliances during orthodontic treatment, especially in complex cases. Therefore, this trial aimed to compare the level of OHRQoL between severe crowding patients receiving clear aligners or fixed appliances within one year of follow-up using a validated self-administered questionnaire (OHIP-14 tool). The hypothesis to be tested was that there were no differences in the level of OHRQoL between severe crowding patients treated with clear aligners and those treated with fixed appliances during a one-year follow-up.

## Materials and methods

Study design, registration, and ethical considerations

A single-centered, two-arm parallel-group randomized controlled clinical trial was conducted at the Department of Orthodontics, University of Damascus Dental School. It was registered in the ClinicalTrials.gov database (Identifier: NCT04866238) and was funded by the University of Damascus Dental School Postgraduate Research Budget (Ref no. 841972995DEN). The protocol of this trial was reviewed by the Local Research Ethics Committee at the University of Damascus Dental School, Syria. The formal approval was obtained before the recruitment process began (Approval no. UDDS-5104-2019PG).

Sample size calculation

Because of the different variable scales, the sample size was calculated twice using Minitab (version 17, Minitab, LLC, State College, PA), and the intended test was a 'two-sample t-test'. The smallest difference requiring detection in the first calculation (for the overall OHIP scores) was 7 points, whereas the second calculation was based on detecting a 1-point difference in each domain. The second calculation was adopted for this trial because of the greater number of patients required in each group, and the following assumptions were used to calculate it: the smallest difference requiring detection for each domain of the OHIP-14 was 1 point (on a combined Likert scale ranging from 0 to 8 for each domain), a significance level of 0.05, a power of 80%, and a standard deviation of 1.02 [27]. It was found that a sample of 18 patients was required for each group. No allowance for loss to follow-up was made. The intention to treat (ITT) analysis was intended in case of patients' attrition of withdrawals.

Participants, eligibility criteria, and setting

Thirty-six adult patients (19 females, 17 males) who had severe crowding required orthodontic treatment with first premolars extraction were recruited from the Department of Orthodontics, University of Damascus Dental School, Damascus, Syria, from February 2019 to March 2020. The patients were randomly assigned to the Clear aligners (CA) group and fixed appliances (FA) group with a 1:1 allocation ratio. All the patients fulfilled the following criteria: (1) Age ranged from 18 to 25 years, (2) Class I malocclusion with severe crowding (more than 5 mm of tooth size-arch length discrepancy) and a score of 25 points and above when using the American Board of Orthodontics Discrepancy Index (ABO-DI) which indicated severe complexity of the case, (3) No congenitally missing or extracted teeth (except for the third molars), (4) No history of previous trauma to the maxillofacial region or surgical interventions. Patients with the following criteria were excluded: (1) Previous orthodontic treatment, (2) Patients with psychological abnormalities, (3) Patients with systematic diseases, and (4) Patients who have known allergies to latex and plastic. Information sheets were distributed to all patients, and informed consent forms were collected. The first premolars were extracted in both groups one week before starting the active orthodontic treatment. No changes in the methods were made following trial registration.

Randomization and blinding

Randomization was performed by one academic staff not involved in this research project. A computer-generated list of random numbers was exported by Minitab (version 17, Minitab, LLC, State College, PA) with an allocation ratio of 1:1. The allocation sequence was concealed using sequentially numbered, opaque, sealed envelopes opened only after the completion of premolars extraction. Patients were sequentially allocated to the treatments in the order they were recruited. Blinding of personnel and participants were not possible; therefore, blinding was only confined to the outcome assessor.

Clear aligners (CA) Group

Treatment was conducted by one single trained examiner (S.T.J.), and parameters were recorded by the same investigator. The virtual setup was prepared using OrthoanalyzerTM software (3Shape, Copenhagen, Denmark). After the first premolars were removed, proper alignment of the case was done, then the attachments were added; after that, the treatment plan was subdivided into subsets considering the maximum transitional movement and rotation for each tooth per aligner 0.25 mm and 3 degrees, respectively. Virtual models were exported using the same 3shape software, printed using 3D validated printer (Moonray; Sprintray, Los Angeles, CA) [28], and clear aligners were fabricated for each model using the 0.762 mm Taguls™ premium (Taguls, Vedia solutions, Mumbai, India) aligner sheets and the Biostar® (Biostar, Scheu-dental, Iserlohn, Germany) thermoforming device. Each aligner was trimmed at the cervical margin of each tooth, polished, disinfected, and packed, then delivered to the patients. During the first appointment for aligners application, the attachments were bonded, and the patients were instructed how to use the aligners and to wear them 20-22 hours, seven days a week. No buttons, interarch elastics, or any additional auxiliaries were used during the observation period in both groups. Each aligner was changed every two weeks. Malocclusion was corrected with no over-correction introduced. Refinements aligners were provided if needed. Each patient was seen every 4-8 weeks (2-4 aligners) to check for aligner fit, attachment stability, and cooperation.

Conventional fixed appliance (FA) group

Conventional orthodontic treatment with fixed buccal appliances was applied to patients in this group by the same examiner (S.T.J.). Fixed appliances were bonded one week following the first-premolars extraction. An MBT prescription brackets with a 0.022-in. slot height (Master Series®, American Orthodontics™, Sheboygan, WI) and anchorage devices (transpalatal arch with a Nance button) were used. The archwire sequence used was: 0.014-inch NiTi, 0.016-inch, 0.016 X 0.022-inch, 0.017 X 0.025-inch NiTi, and finally 0.019 X 0.025-inch stainless steel wire [29]. The patients in this group were seen every 3-4 weeks to monitor the treatment process and make the required activations and modifications during treatment. Archwires were replaced when crowding started to decrease, and the insertion of the following archwire appeared to be possible without applying excessive force on the engaged teeth. The treatment was considered finished when the complete alignment of the teeth was done, extraction spaces were completely closed, and the American Board of Orthodontics (ABO) guidelines were achieved.

Outcome measure: oral-health related quality of life

The OHIP-14 questionnaire was employed in this trial to measure the OHRQoL. This tool has been evaluated in previous reports and was found valid and reliable [26,30]. The validated Arabic version was used in this study [31]. It consisted of 14 items covering seven domains, and each domain contained two questions [27]: functional limitation, physical pain, psychological discomfort, physical disability, psychological disability, social disability, and handicap. Patients were asked to express how they experienced an oral health impact. Each item was scored on a 5-point Likert scale: 0, never; 1, hardly ever; 2, occasionally; 3, fairly often; and 4, very often or every day. The scores in each domain ranged from zero to eight. The overall OHIP-14 scores ranged from 0 to 56. A higher OHIP-14 score indicates poorer OHRQoL. Patients were given the first questionnaire before starting the active orthodontic treatment with FA and CA, which was considered the baseline (T0). Then the questionnaires were given at various times during comprehensive orthodontic therapy: 1 week (T1), two weeks (T2), one month (T3), six months (T4), and 12 months (T5) after starting the active orthodontic treatment.

Statistical analysis

SPSS program (version 21, IBM Corp., Armonk, NY) was used for data analysis. Compatibility between the two groups according to gender, age, and discrepancy index (DI) scores distribution was tested using a two-sample t-test, chi-square test, and Mann-Whitney U tests, respectively. After applying the Anderson-Darling Normality test, nonparametric tests were used to explore the changes in OHRQoL since the data did not follow the normal distribution. The Mann-Whitney U test was used to detect statistically significant differences between the two groups at each assessment time. Friedman test was used to test the significant differences in OHIP-14 scores during the study period in each group separately. The level of significance was set at 5%.

## Results

**Figure 1:**
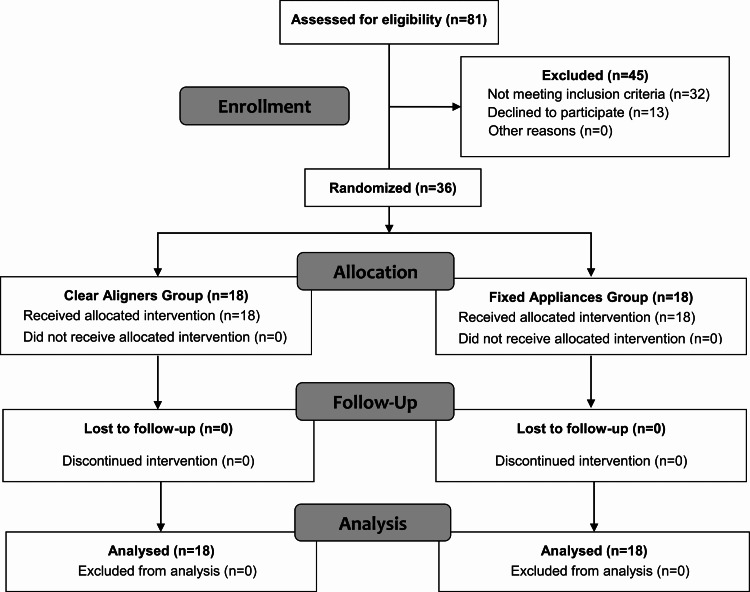
Consolidated Standards of Reporting Trials (CONSORT) flow diagram of patients' recruitment and follow-up.

A total of 36 orthodontic patients met the inclusion criteria and were enrolled. Figure 1 shows the CONSORT flow diagram of patients’ recruitment and follow-up. Eighteen patients received treatment with clear aligners (nine males and nine females) and 18 with fixed appliances (eight males and 10 females). The patients in the FA group were on average 20.86±1.98 years old. The CA patients were on average 21.27±1.87 years of age. The mean DI scores were similar between the CA and FA groups, with no statistically significant difference between them (P=0.702). All the patients completed the questionnaires at all observational time points without missing data. During the first year of treatment (i.e., the follow-up period of the current study), no one of the included patients completed the whole treatment or lost to follow-up. The demographic information of participants is displayed in Table 1.

**Table 1:** Basic sample characteristics (Age, Gender, and DI score) *CA: Clear Aligners group, ** FA: Fixed Appliance group, † DI: Discrepancy index ‡  Employing chi-square test, § Employing two-sample t-test,  ‖ Employing Mann–Whitney U test.

Variable		CA group* n=18	FA group** n=18	Both groups n=36	P-value
Gender	Male	9 (50%)	8 (47.1%)	17 (47.2%)	0.672 ‡
	Female	9 (50%)	10 (52.7%)	19 (52.8%)	
Mean age (SD)		21.27 (1.87)	20.86 (1.98)	21.03 (1.96)	0.982 §
DI† scores (SD)		26.89 (1.45)	27.22 (2.11)	27.05 (1.76)	0.702 ‖

For the psychological discomfort, psychological disability, social disability, and handicap, there were no significant differences between the CA and FA groups (P˃0.05) at all assessment times except for the psychological disability at T1, where a statistically significant difference between the two groups was observed (P=0.008), with the patients in the FA group recording higher mean scores in comparison to patients in the CA group. There were significant differences (P˂0.001) between the assessment times when testing intergroup comparisons (Table 2).

**Table 2:** Descriptive statistics of the scores of the seven OHIP-14 domains and the results of significance tests. ꝉ Friedman two-way ANOVA was used to test the significant difference in OHIP-14 scores during the study period. ‡ Mann–Whitney U test was used to detect statistically significant differences between the two groups at each assessment time. *Significant at P˂0.05.  CA: Clear aligners group, FA: Fixed appliances group. T0: Baseline, T1: 1 week, T2: 2 weeks, T3: 1 month, T4: 6 months, T5: 12 months after starting the active orthodontic treatment

Domain	Time	CA			FA			CA vs FA	
		Mean (SD)	Median	P-value ꝉ	Mean (SD)	Median	P-value ꝉ	Mean Difference	P-value ‡
Functional Limitation	T0	0.06 (0.24)	0.00	<0.001*	0.06 (0.24)	0.00	<0.001*	- 0.00	≈ 1.000
	T1	1.47 (1.33)	1.00		4.41 (1.66)	4.00		- 3.00	<0.001*
	T2	0.88 (1.11)	0.00		3.24 (1.35)	3.00		- 3.00	<0.001*
	T3	0.53 (0.80)	0.00		2.35 (1.41)	3.00		- 2.00	<0.001*
	T4	0.59 (0.80)	0.00		1.59 (1.12)	1.00		- 1.00	0.011*
	T5	0.24 (0.44)	0.00		0.82 (0.95)	1.00		0.00	0.085
Physical Pain	T0	0.29 (0.47)	0.00	<0.001*	0.71 (1.69)	0.00	<0.001*	0.00	0.718
	T1	3.59 (1.54)	4.00		5.12 (1.45)	5.00		- 2.00	0.007*
	T2	2.00 (1.28)	2.00		3.77 (1.35)	4.00		- 2.00	0.001*
	T3	1.77 (1.20)	1.00		3.71 (1.93)	4.00		- 2.00	0.004*
	T4	1.18 (1.13)	1.00		2.71 (1.45)	2.00		- 2.00	0.003*
	T5	0.59 (0.80)	0.00		1.82 (1.43)	2.00		- 1.00	0.007*
Psychological Discomfort	T0	1.59 (1.42)	2.00	<0.001*	2.12 (2.25)	2.00	<0.001*	- 0.00	0.642
	T1	1.53 (1.84)	1.00		1.94 (2.16)	1.00		- 0.00	0.547
	T2	1.00 (1.50)	0.00		1.53 (1.88)	1.00		- 0.00	0.318
	T3	0.82 (0.95)	1.00		1.41 (1.73)	1.00		- 0.00	0.558
	T4	0.65 (1.12)	0.00		1.00 (1.17)	1.00		0.00	0.335
	T5	0.65 (1.06)	0.00		0.77 (0.97)	0.00		0.00	0.667
Physical Disability	T0	0.65 (0.93)	0.00	<0.001*	0.88 (1.69)	0.00	<0.001*	- 0.00	0.890
	T1	2.47 (1.98)	3.00		3.82 (1.91)	4.00		- 1.00	0.040*
	T2	1.29 (1.26)	1.00		2.59 (2.03)	3.00		- 1.00	0.049*
	T3	0.82 (0.95)	1.00		2.41 (1.84)	3.00		- 2.00	0.013*
	T4	0.41 (0.87)	0.00		1.65 (1.62)	1.00		- 1.00	0.007*
	T5	0.24 (0.44)	0.00		1.12 (0.93)	1.00		- 1.00	0.006*
Psychological Disability	T0	1.29 (1.26)	1.00	<0.001*	2.00 (1.62)	2.00	0.001*	- 1.00	0.215
	T1	1.18 (1.43)	1.00		2.82 (2.07)	2.00		- 2.00	0.008*
	T2	0.94 (0.97)	1.00		1.88 (1.62)	1.00		- 1.00	0.068
	T3	0.77 (0.75)	1.00		1.53 (1.66)	1.00		0.00	0.318
	T4	0.59 (0.71)	0.00		1.00 (1.17)	1.00		- 0.00	0.399
	T5	0.41 (0.51)	0.00		0.77 (0.97)	0.00		0.00	0.449
Social Disability	T0	1.94 (1.60)	1.00	<0.001*	2.41 (2.15)	2.00	<0.001*	0.00	0.618
	T1	1.65 (1.32)	1.00		3.00 (2.18)	3.00		- 1.00	0.071
	T2	1.12 (0.93)	1.00		2.06 (1.75)	2.00		- 1.00	0.079
	T3	0.82 (0.81)	1.00		1.59 (1.73)	1.00		- 1.00	0.174
	T4	0.65 (0.70)	1.00		1.35 (1.27)	1.00		- 1.00	0.109
	T5	0.59 (0.62)	1.00		1.00 (1.00)	1.00		0.00	0.294
Handicap	T0	2.06 (2.11)	1.00	<0.001*	1.65 (1.80)	1.00	<0.001*	0.00	0.654
	T1	1.18 (1.55)	1.00		1.71 (1.96)	1.00		0.00	0.630
	T2	0.47 (0.94)	0.00		1.41 (1.94)	1.00		- 0.00	0.134
	T3	0.29 (0.77)	0.00		1.12 (1.87)	0.00		0.00	0.209
	T4	0.18 (0.39)	0.00		0.77 (1.20)	0.00		- 0.00	0.191
	T5	0.12 (0.33)	0.00		0.59 (0.80)	0.00		- 0.00	0.121
Overall	T0	7.82 (4.35)	7.00	<0.001*	9.94 (7.22)	8.00	<0.001*	- 1.00	0.502
	T1	12.94 (7.54)	12.00		22.88 (9.60)	21.00		- 10.00	0.002*
	T2	7.71 (5.68)	6.00		16.41 (9.27)	14.00		- 8.00	0.001*
	T3	5.82 (3.96)	4.00		14.12 (9.07)	10.00		- 6.00	0.001*
	T4	4.12 (3.18)	4.00		10.12 (6.84)	9.00		- 5.00	0.001*
	T5	2.88 (2.57)	3.00		6.88 (3.81)	7.00		- 4.00	0.003*

In the CA and FA groups, the patients reported a significant increase (P<0.001) in the functional limitation one week following appliance insertion (T1). A significant decrease was observed at the subsequent assessment times; nevertheless, the scores were still significantly poorer than recorded at the pre-treatment assessment time. At the following assessment times, there were significant differences between the two groups (P˂0.05) except for the difference after one year (T5), which was insignificant (P=0.085). 

Concerning physical pain, a significant increase (P˂0.05) was reported in both groups one week after the treatment had started (T1). Over time, gradual improvements in patients' assessments were recorded. However, the differences were significant during all assessment times in the CA and FA groups compared to the data obtained at the baseline (T0). Intergroup differences were significant (P˂0.05) concerning physical pain at T1, T2, T3, and T4. For the physical disability, there were significant differences (P<0.05) between the two groups after appliances’ placement at all assessment times. Scores were higher in the FA group than those in the CA group. The highest scores were recorded in the CA and FA groups at T1 (mean values of 2.47±1.91, 3.82±1.91, respectively).

Concerning the overall OHIP-14, after one week of treatment, the scores significantly increased (P˂0.001) in comparison to T0 and reached their peak (12.94 for the CA group, 22.88 for the FA group). After two weeks, the overall score in both groups decreased (P˂0.001), with the patients in the CA group recording an approximate mean score (7.71±5.68) to the baseline score (7.82±4.35). After one year of treatment (T5), both groups reached an overall score lower than that recorded at the baseline (T0). After starting the active treatment, there were significant differences (P˂0.05) between the two groups at all assessment times. 

## Discussion

The modern dental practice relies much more attention on patients' preferred preferences of their oral health status. OHRQoL is a conception that includes individual evaluation of perceived physical, psychological, and social aspects of oral health. This concept should be a part of the evaluation of oral health status in patients seeking orthodontic treatment [32]. When evaluating the available literature, it seems to be that this is the first study that assesses the role of in-office clear aligners on OHRQoL during the treatment of severe crowding cases in comparison with the traditional fixed appliances.

By convention, and according to the clinical expertise of two senior researchers in this trial, a difference of one point in any of the OHIP-14 domains or of 7 points in the overall OHIP-14 score was considered clinically significant in the interpretation of the current results. At the baseline, the mean scores of all domains of the OHIP-14 questionnaire were similar between the fixed appliance and clear aligner groups. After the commencement of treatment, significant changes in OHRQoL were observed. During the first week of treatment, the highest deterioration in the OHRQoL occurred. The overall score significantly increased and reached its peak in both of the studied groups indicating that OHRQoL was poorer in the fixed appliance group than in the clear aligner group, with a mean difference of 10 points. During the second week, the levels of OHRQoL improved moderately. The overall score significantly decreased in both groups, with a higher mean score (i.e., worse OHRQol) in the FA group compared to the CA group. After that, the scores continued to decline until the final assessment point (T5), where the mean scores were 2.88±2.57 and 6.88±3.81 in the CA and FA groups, respectively, indicating that the QoL became better than what was recorded at baseline. These findings agree with the findings of Chen et al., who reported that the QoL was at its worst point one week after fixed appliances’ insertion, and that was because the combination of physical pain, psychological discomfort, and physical disability were at their highest levels [16].

Clinically significant differences between the two groups were found in four domains: functional limitation, physical pain, physical disability, and psychological disability (for the latter domain, the CA group was significantly better than the FA group at the first-week assessment only). Both groups had similar mean scores during the follow-up period for the other three domains.

These findings indicate that patients treated with fixed appliances suffered from adverse side effects more than those treated with clear aligners like pronunciation difficulties, taste sensation weakness, eating difficulty, diet unsatisfaction, and meal interruption.

According to these, eating was the most significant challenge to OHRQoL for patients treated by fixed appliances, whereas the clear aligners produced fewer chewing and eating disturbances during treatment. This could be attributed to the removability of clear aligners during eating and chewing, and the comfortable structure of the clear aligners due to their smooth surfaces and less bulkiness [6]. Therefore, clear aligners do not interfere with the intraoral soft tissues during various functional movements, which explains their less functional limitation compared to fixed appliances.

For the pronunciation difficulties, the results of this study indicated that the patients in the FA group suffered from problems more than those in the CA group during the first six months of treatment. These findings were different from the findings of Melo et al. indicated that the orthodontic aligners caused more speech difficulties than the fixed appliances immediately and three days after insertion of the appliances with no significant differences after 30 days of treatment [33]. These differences could be attributed to the fact that the anchorage control with the labial fixed appliances was maintained using TPAs and Nance buttons in the current study, which could be the leading cause of speech distortions, especially in the initial stages of treatment [34]. On the other side, the clear aligners can be removed from the mouth for a limited period during social situations, and that could reduce the disturbances during pronunciation. Furthermore, the first assessment point in this study was 1-week after the treatment had started, allowing the patients to adapt to their aligners, which might be reflected in the questionnaire results.

Physical pain is considered a major factor affecting the OHRQoL of patients undergoing orthodontic treatments [26]. This was significantly less in the CA group than the FA group during all assessment times after the onset of treatment. The results of this study go in line with the findings of Gao et al. [26] and AlSeraidi et al. [35], who found that the pain levels accompanied by the fixed appliances were greater than that with the clear aligners during the initial phase of treatment. In contrast, these results differ from those in Shalish et al. study (which used a scale from 1 to 10 to assess the pain for the first week and on the 14th day) that found that the patients treated with lingual appliances and clear aligners presented higher levels of pain compared with those treated with fixed appliances during the study period [24]. The lower pain reported by CA patients could be because it is a removable appliance. Generally, removable appliances are less painful than fixed appliances [36], because fixed appliances produce higher levels of tension, pressure, pain, and sensitivity of teeth, unlike clear aligners with intermittent force application allowing tissue reorganization before compressive forces are reapplied [37].

In the aspect of physical disability, the result of this study was not surprising since aligner patients had no eating limitations after taking off their appliances, whereas patients in the fixed group had chewing difficulties and many concerns when eating hard food due to the possible bracket detachments. When the teeth start to move, inflammatory mediators, like substance P, have been shown to be present in the periodontal ligament (PDL) [38], which sensitize the nociceptors scattered in this region [39]. These sensitized nociceptors compress under chewing, stimulating a more painful signal than at rest. Importantly, this phenomenon was only evident in the FA group due to the continuous nature of the forces used, and these forces sensitized the nociceptors in the PDL of the patients treated in the FA group more than the patients treated in the CA group, making them more susceptible to variations in compression of the PDL.

Limitations

The current study has some potential limitations. One of these limitations is the absence of any interim assessment of OHRQoL during the first few days following appliance first wear (CA) or bonding (FA). Therefore, the findings were confined to the evaluation for seven days only. Second, the duration of the follow-up was only one year, although the overall treatment time could reach 18-24 months on average. The complete picture of the impact of these two treatment modalities on the OHRQoL is not given. Future studies might consider the comparison between in-house clear aligners and aligners fabricated by well-known companies in terms of their effects on patient-oriented outcome measures.

## Conclusions

During the early phase of treatment, the greatest deterioration in OHRQoL occurs. With ongoing treatment, OHRQoL gradually improved. Patients treated with clear aligners had less impact on OHRQoL than those treated using conventional fixed appliances during the first year of treatment. Functional limitation, pain, and physical disability were the most affected aspects during orthodontic treatment in both groups. 
